# Neuroimaging in Internet gaming disorder comorbid with Attention-deficit disorder

**DOI:** 10.1192/j.eurpsy.2023.1931

**Published:** 2023-07-19

**Authors:** B. Wildenberg, R. Pires, D. Pereira, I. Faria, N. Madeira

**Affiliations:** 1 Psychiatry, Centro Hospitalar e Universitário de Coimbra; 2 Institute of Psychological Medicine; 3 Neurorradiology, Centro Hospitalar e Universitário de Coimbra, Coimbra, Portugal

## Abstract

**Introduction:**

Internet gaming disorder (IGD) refers to a pattern of persistent gambling behaviour or recurring gambling, on the Internet, with impaired control, increased priority, and continuation or escalation of gambling despite the occurrence of negative consequences. Currently, online gambling - more frequent among men - represents a very important, constantly growing economic activity. Since the COVID-19 pandemic and the need to conduct classes online, there has been an increase in the rate of IGD in children and youth (7-25 years old). Comorbidities, namely attention deficit hyperactivity disorder (ADHD), have been associated with a higher prevalence of IGD. They share core traits, such as impulsiveness, seeking immediate reward, deficient motivation, and hostility, which may translate into neuroimaging similarities.

**Objectives:**

Non-systematic review of the literature about neuroimaging of IGD comorbid with ADHD.

**Methods:**

A search was conducted on PubMed and other databases, using the MeSH terms “Internet Addiction Disorder”, “Attention Deficit Disorder with Hyperactivity” and {“MRI” OR “fMRI” OR “functional connectivity” OR “neuroimaging OR neural alteration OR neuronal alteration OR neural change"}.

**Results:**

IGD and ADHD have shared and disorder specific patterns of structural and functional abnormalities, particularly in reward function. For instance, IGD has been associated with lower putamen grey matter volume (GMV), while ADHD patients have lower GMV in the orbitofrontal cortex. Disorder-specific fMRI activation has been observed in the precuneus in IGD; in ADHD, there is special activation in the fusiform gyrus. Finally, shared structural and functional alterations between IGD and ADHD seem to converge in the prefrontal-striatum circuit, especially the anterior cingulate cortex.

**Conclusions:**

ADHD has been suggested as the most significant predictor of IGD in cross-sectional and prospective studies, however there is no study that clarifies their relationship. It is unclear whether IGD causes ADHD symptoms or whether a problem with gambling is a prodromal sign of the development of full ADHD. Studies revealing common neurobiological foundations between these disorders are pivotal to understand their basic mechanisms, while alerting to the necessity to screen for both pathologies when one is present. They may also point to an overlapping target (the reward circuit) for behavioural and pharmacological treatment.

**Disclosure of Interest:**

None Declared

